# Community Empowerment and Mentality as a Treatment in Treatment-Resistant Trichotillomania: A Case Report

**DOI:** 10.7759/cureus.70163

**Published:** 2024-09-25

**Authors:** Kimberly A Kluglein, David F Lo, Don Shamilov, Christian P White

**Affiliations:** 1 College of Osteopathic Medicine, Nova Southeastern University Dr. Kiran C. Patel College of Osteopathic Medicine, Clearwater, USA; 2 Medicine, American Preventative Screening& Education Association, Stratford, USA; 3 Department of Medicine, Rowan-Virtua School of Osteopathic Medicine, Stratford, USA; 4 Department of Psychiatry, Rowan-Virtua School of Osteopathic Medicine, Stratford, USA

**Keywords:** community support, positive mentality, self-inflicted hair loss, treatment-resistant, trichotillomania

## Abstract

Trichotillomania, a psycho-dermatologic disorder defined by self-inflicted often noticeable hair loss through hair-pulling, is difficult to manage and treat. Its etiology is multifactorial and frequently is complicated by comorbid conditions like anxiety and depression. Evidence-based treatment is limited but options involve a combination of pharmacotherapy and cognitive-behavioral therapy (CBT), habit reversal training (HRT), and acceptance and commitment therapy (ACT). Many individuals still struggle with the condition despite therapy efforts. This is the case of a 24-year-old female who has a diagnosis of treatment-resistant trichotillomania and found remission of hair-pulling and subsequent hair regrowth by a combination of mindset reframing, physical barriers, and community support. Through these mechanisms, she has managed to stay pull-free for 9 months, the longest period she has experienced without hair-pulling episodes in 10 years. This case demonstrates that novel approaches using enhanced mindsets and shared experiences can provide positive results for people affected by trichotillomania. The implications of this case may help other individuals suffering from this diagnosis achieve remission of hair-pulling. Additionally, this case serves as a catalyst for future research to be done into the long-term effects of community empowerment and mentality-based interventions as treatments for trichotillomania.

## Introduction

Trichotillomania, a complex psycho-dermatologic disorder and form of traumatic alopecia listed in the Diagnostic and Statistical Manual of Mental Disorders (DSM-5) under the category of obsessive-compulsive and -related disorders, is characterized by recurrent hair-pulling resulting in noticeable hair loss, distress, and impairment in various areas of functioning [1-2]. Despite its prevalence and impact on individuals’ quality of life, trichotillomania remains relatively understudied compared to other psychiatric conditions [3]. Additionally, the disorder shares overlapping features with impulse control disorders, mood disorders, and anxiety disorders, further complicating its diagnosis and management [4]. Clinically, trichotillomania presents with an array of symptoms, ranging from mild to severe hair-pulling behavior. While many of the recent case reports published on trichotillomania have focused on acceptance and commitment therapy (ACT) [5] and habit reversal training (HRT) [6] for remission of hair-pulling, our case focuses on the positive effects of community support, empowerment, mindset, and the use of technology as modalities for treatment. This case report aims to contribute to the growing literature on trichotillomania by providing a detailed analysis of a patient’s journey, including their initial presentation, diagnostic evaluation, treatment course, and outcomes. By elucidating the complexities of this disorder, we hope to enhance clinicians’ understanding, improve diagnostic accuracy, and refine treatment strategies for individuals affected by trichotillomania.

## Case presentation

This case follows the story of a 24-year-old White female with a past medical history of anxiety, depression, dermatillomania, and treatment-resistant trichotillomania who first began hair-pulling when she was 14 years old and became known to us in 2023. During the first 2 years with her condition, she experienced shame and confusion about why she felt the need to pull her hair and did not know what to do about it. At 16 years old, she discovered trichotillomania and disclosed it to her psychiatrist but not to her dermatologist. The diagnosis of trichotillomania was made by clinical assessment and additional diagnostic tests were unnecessary and thus not performed. She saw multiple therapists, with whom she felt more comfortable discussing her condition. From ages 16 to 22, she tried cognitive-behavioral therapy (CBT), and HRT, saw multiple providers and tried numerous selective serotonin reuptake inhibitors (SSRIs); however, neither medication nor weekly therapies improved her condition and were therefore discontinued. Records could not be obtained to confirm the exact dosages of medications tried and failed. Currently, with her new treatment plan initiated in early 2023, she has been pull-free for 9 months with significantly reduced pulling sessions for nearly 2 years and celebrates this accomplishment.

The patient attributes a large part of her recovery to the aspect of her treatment plan that includes adopting a positive mindset in regard to her feelings about trichotillomania. Some of the strategies she has used to promote a positive mindset include reshaping the way she views aspects of her diagnosis. Where she previously saw regrowth as a sign of her struggle and felt shame, she reframed how she views regrowth as a sign of progress instead. Another factor has been abandoning her self-entitled, indifferent mindset of self-sabotage in favor of a more productive outlook with less destructive behaviors. She does this by verbalizing her intentions not to pull and setting strong limits with herself for pulling. Further changing her outlook, she has given herself grace in her journey. Recognizing that trichotillomania is not a disease that is easy to treat has helped her as she is learning to forgive herself, reducing blame and guilt, and has been kind to herself, as she once was not.

Aside from reframing her mindset, she has also found physical steps she can take not only for her to stop pulling but for her hair to grow healthy. The patient finds that wearing a beanie significantly reduces her pulling and has helped her during particularly challenging times. Exercise has also had a positive impact as it helps reduce her stress and anxiety, which often manifests as episodes of hair-pulling.

Her treatment also involves the use of online resources, which have been extremely helpful for her. In May 2023, she joined an online support group for trichotillomania and began conversing and sharing her story with others in the community. Having other people going through the same struggle as she is and being able to talk with them has made a big difference in her continued abstinence from pulling. Along with the support group, she is using an application on her mobile phone called “I am Sober,” [7] as a tool to hold her accountable and track her progress.

For hair regrowth, the patient finds supplementing biotin has been important for her hair regrowth journey to be healthy and strong. In addition, she tends to use gentle shampoos formulated for sensitive scalps, as her scalp health has suffered during episodes of hair-pulling. Essential oils like lavender and rosemary have helped her to keep her scalp in optimal condition for hair regrowth and have reduced inflammation.

**Figure 1:**
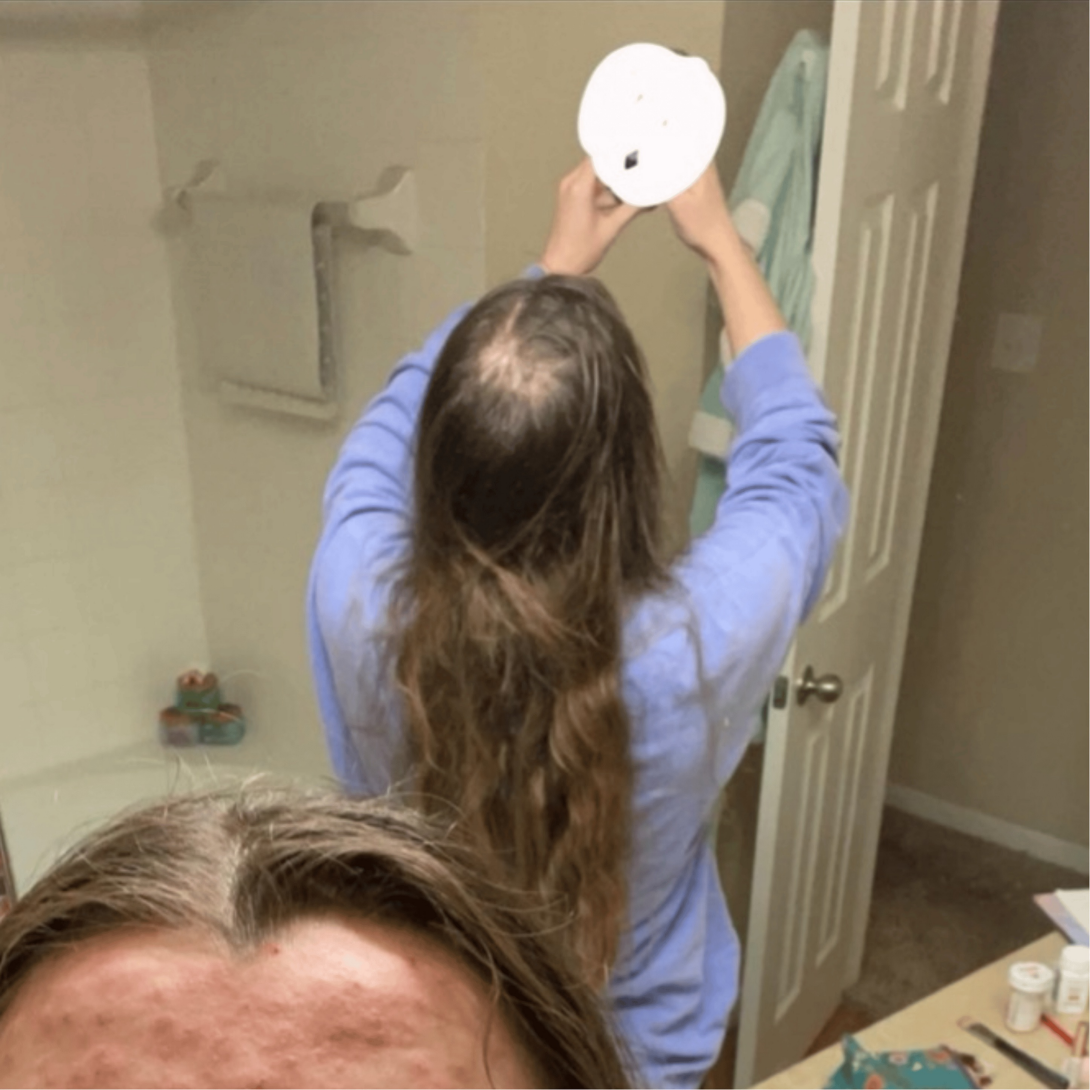
Patch of self-inflicted hair loss of the mid-parietal scalp, mirror view

**Figure 2:**
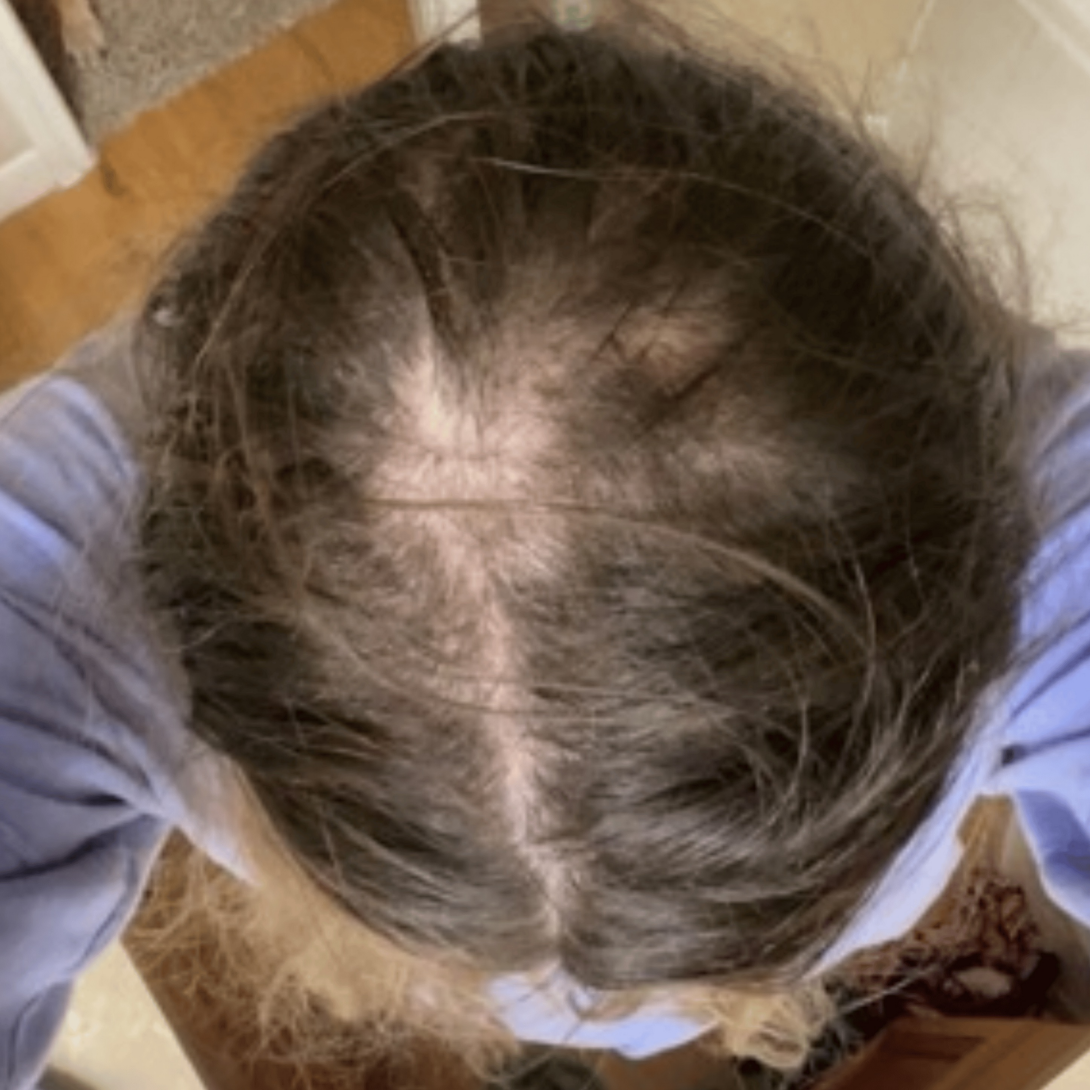
Patch of self-inflicted hair loss of the mid-parietal scalp, overhead view

**Figure 3:**
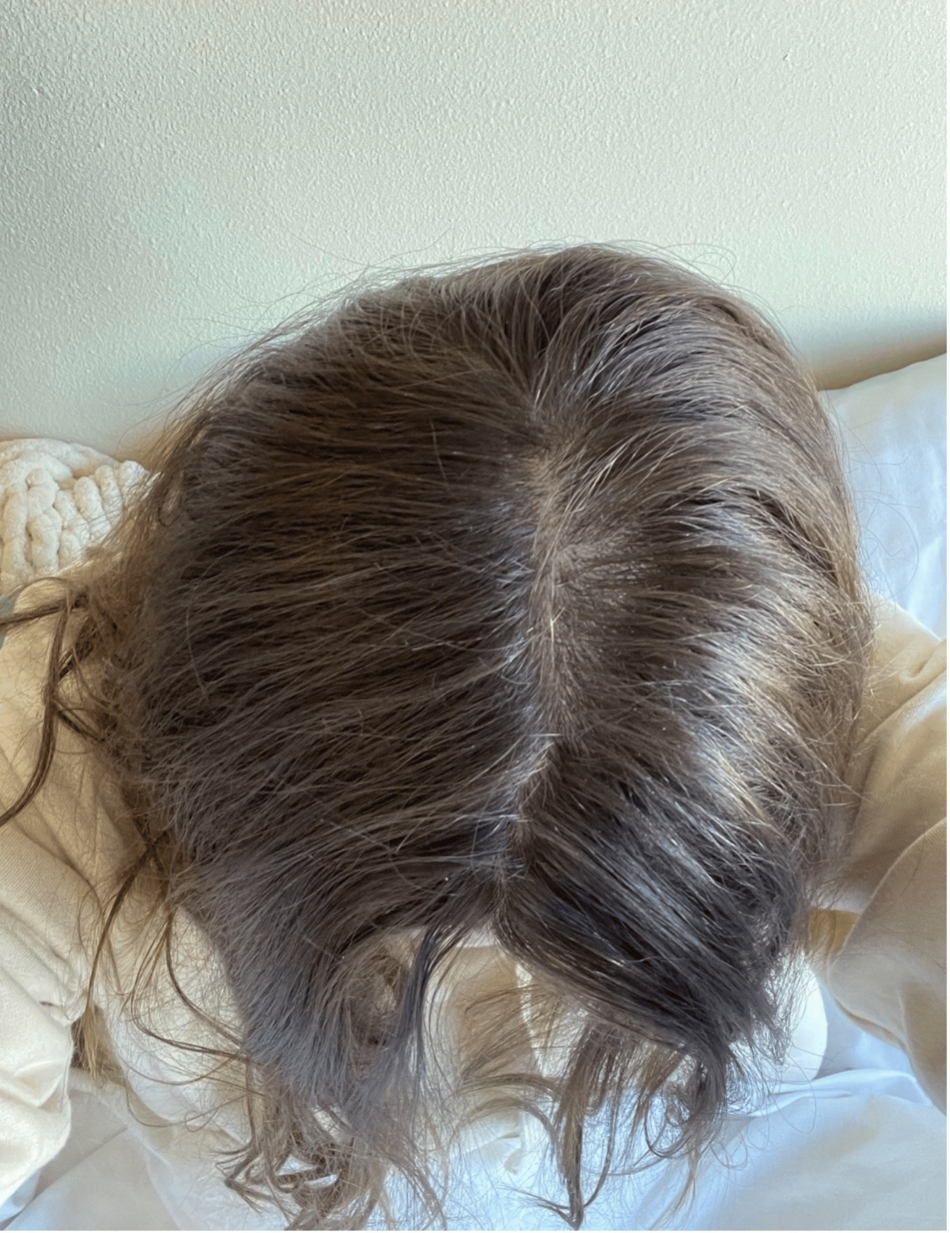
Hair growth 7 months into total remission of hair-pulling episodes, overhead view

**Figure 4:**
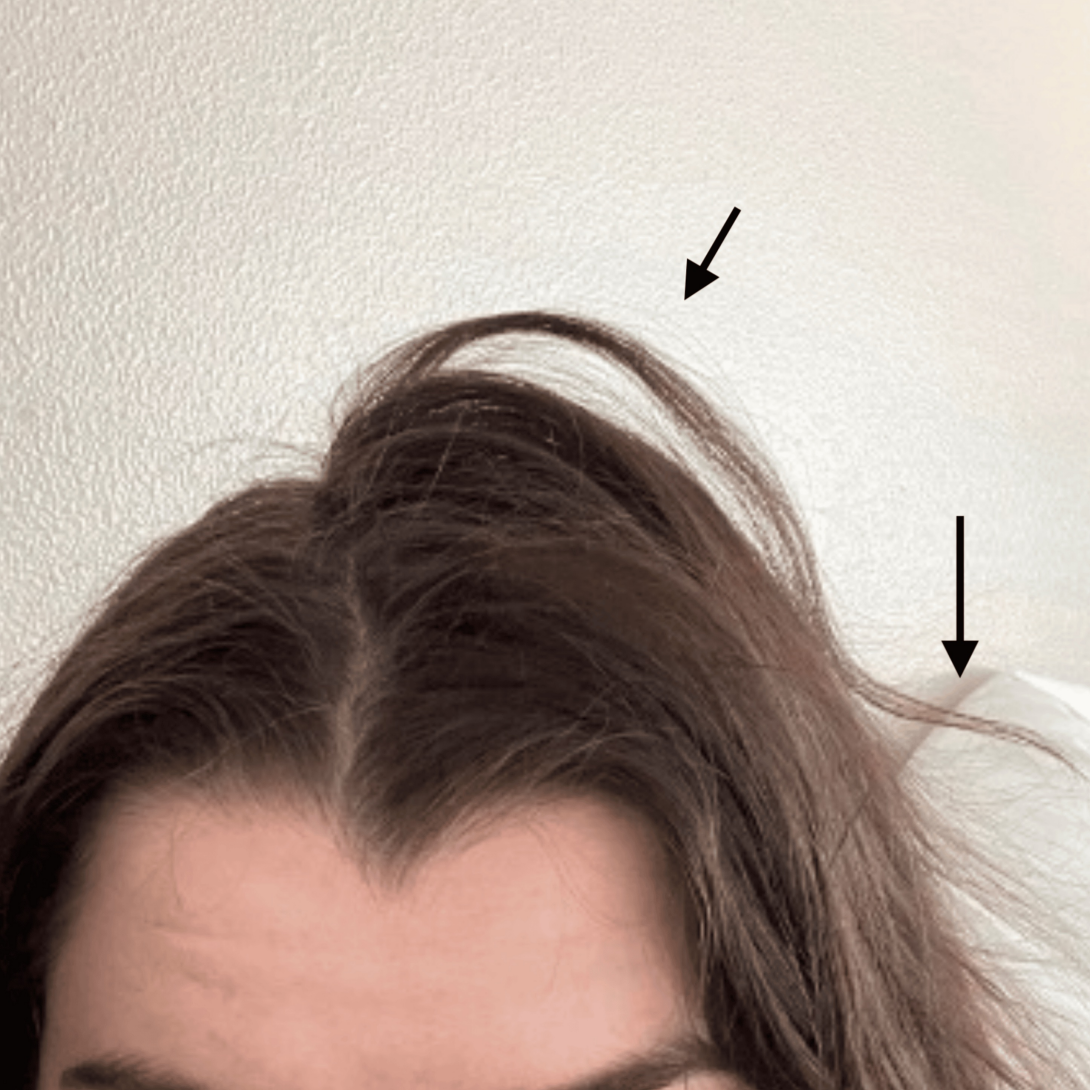
Hair growth 7 months into total remission, highlighting an area of hair she has not pulled in approximately 16 months

This patient has been keeping track of her hair regrowth journey by taking photographs, as shown in Figures 1-4. There were approximately 7 months between Figure 1 and Figure 2 to Figure 3 and Figure 4. Figure 4 shows an area of hair that she has not pulled for approximately 16 months, highlighting the impact of reduced pulling sessions. With the aforementioned treatment methods employed, she experienced the fastest time to remission and the longest period of pull-free episodes since she first started hair-pulling 10 years ago. This outcome has been assessed clinically by evidence of full-volume hair free of broken strands and other evidence of pulling in contrast to her initial assessment. Subjective assessment was also employed as she reports this is the most progress she has had since her diagnosis.

## Discussion

The etiology of trichotillomania is multifactorial, involving a combination of genetic, neurobiological, psychological, and environmental factors [2,8,9]. Psychosocial stressors, trauma, and learned behaviors also contribute to the development and maintenance of trichotillomania symptoms [10]. Additionally, comorbid psychiatric conditions, such as depression, anxiety, and body dysmorphic disorder, are common among individuals with trichotillomania, further complicating clinical management [11,12].

Diagnosis of trichotillomania can be complicated as some patients are not forthcoming about their habits due to fear of judgment and shame [13,14] or are in denial about their condition. Individuals may experience varying degrees of insight into their condition; some may acknowledge the behavior as problematic while others minimize or deny its significance [15]. A thorough physical exam with trichoscopy and scalp biopsy can aid in diagnosis for patients presenting to the dermatologist. On exam, hair around the area of insult is often damaged, skin can be crusted and excoriated from scratching and the pull test is negative [16]. On trichoscopy, hair shafts are fractured, often at different distances, causing the appearance of irregular black dots, coiled or hook hairs, tulip hair, flame hair, V-sign, and hair powder [17]. Biopsy will classically reveal trichomalacia, pigment clumps, peribulbar hemorrhage, hair canal pigment casts, and lack of lymphocytic infiltrates seen in alopecia areata [17].

Despite the significant impact of trichotillomania on individuals’ well-being, [3,15] treatment options remain limited and evidence-based interventions are sparse. Current therapeutic approaches typically involve a combination of pharmacotherapy, CBT, HRT, and ACT [15,18-20]. However, response rates are as low as 25-68% [2] and many individuals continue to struggle with symptoms despite receiving treatment.

This case has the potential to influence the way trichotillomania is approached and treated by both dermatologists and psychiatrists alike. Moreover, it highlights the importance of addressing the psychosocial effects of trichotillomania. To our knowledge, no other cases incorporate the use of mindset reframing and empowerment through shared success within online communities and sobriety apps as a modality of treatment in trichotillomania, making it a novel approach to disease remission. Clinicians should remind patients that trichotillomania is a difficult disease to treat and that failure of treatment is not a reflection of the patient. Patients should be encouraged by providers to join online support groups and utilize sobriety apps as a singular treatment or in addition to other treatment protocols.

Clear implications of this case are the potential for further research into mindset-based interventions as a treatment option for struggling patients. There is a need for longitudinal studies to assess the long-term effectiveness of mindset modification and community support on recovery. Due to the nature of this disease, there are very few proven treatment options for patients and treatment is not a one-size-fits-all, making the findings in this case significant. Patients often fail numerous treatments before finding the one effective for them; however, with these new findings, another option can potentially be added for patients seeking help.

Limitations of this case include the potential for confounding factors in this subject from concurrent depression, anxiety, and dermatillomania, limited generalizability due to individual-specific experience, reliance on subjective reporting, lack of detailed clinical assessment, self-reported outcomes, and short follow-up period for assessing long-term sustainability. There is also a limited evidence base for the effectiveness of reported strategies as there is no objective research on the subject.

## Conclusions

This case report underscores the importance of community support and positive mentality reframing as a treatment for trichotillomania. Healthcare providers should be aware of the clinical signs for diagnosis as many patients may hide their disease and quietly struggle. When one treatment is not working, clinicians should be quick to change their approach to prevent significant morbidity. As evidence-based treatment options are limited in trichotillomania, this novel approach may serve as another option for individuals suffering from treatment-resistant disease. Further research needs to be performed to assess the long-term sustainability and efficacy of these modalities.
